# Supplementary material for: UNSW Face Test: A screening tool for super-recognizers
Source: PLoS One. 2020 Nov 16;15(11):e0241747. doi: 10.1371/journal.pone.0241747 (PMC7668578; doi:10.1371/journal.pone.0241747)
Supplement: S1 Appendix — (DOCX) [file pone.0241747.s001.docx]

**UNSW Face Test: A screening tool for super-recognizers**

**S1 Appendix: Additional demographics analysis**

James D. Dunn, Stephanie Summersby, Alice Towler, Josh P. Davis, and David White

*Corresponding Author: James D. Dunn (*[*j.d.dunn@unsw.edu.au*](mailto:j.d.dunn@unsw.edu.au)*)*

Analysis by participant ethnicity

The effect of ethnicity on accuracy in the UNSW Face Test was analysed using Online Samples 1 and 2. There were small differences in average accuracy between the different ethnic groups. Participants who self-identified as having Asian, Hispanic, Middle Eastern, and mixed ethnicity all had higher average accuracy on the UNSW Face Test than white participants, despite these ethnicities being the minority population in Australia (see Supplementary Figure 1). This pattern of results can be explained if the majority of these other ethnic groups were born in predominantly European countries, as these participants would have critical developmental experience recognising both white and non-white faces that would reduce the own-ethnicity effect (see [1]).


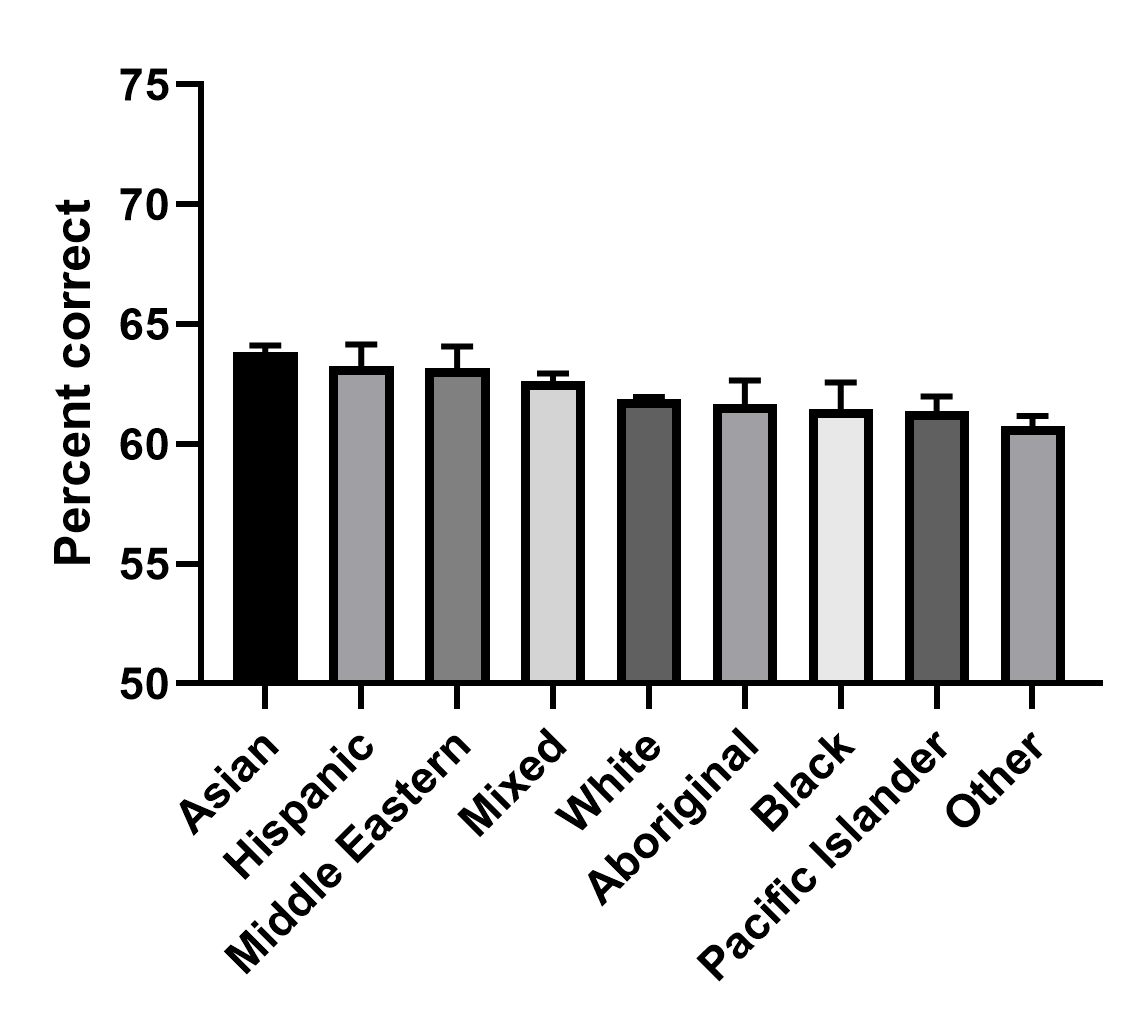


*Supplementary Figure 1. Comparison of group accuracy for different ethnic groups. Error bars denote 95% confidence intervals.*

Differences in accuracy between ethnic groups can be attributed to the Own-Ethnicity Effect (or Cross-Race Effect), whereby individuals are less accurate when identifying faces of a different ethnicity to their own (for review [2]). As the UNSW Face Test includes images of both white and non-white faces, it is important to determine the full extent of the own-ethnicity effect in this test. This analysis was conducted separately for the Normative sample and both online samples combined.

For the Normative sample, we compared the accuracy of white (N = 212) and non-white participants (N = 78) on the white and non-white faces in the test. The analysis was conducted using a 2x2 mixed factor ANOVA with Item Ethnicity (white, non-white) as a within factor and Participant Ethnicity (white, non-white) as the between factor. A significant interaction between factors suggested the presence of an own-ethnicity effect, *F*(1,288) = 5.51, *p* = .020, η_p_^2^ = .019. Simple main effects confirm this, as white participants performed 5.4% worse on non-white face items than the white face items, *F*(1,288) = 75.22, *p* = .000, η_p_^2^ = .207. We also found that the Non-white participants were 2.6% worse on Non-white face items than white face items, *F*(1,288) = 6.32, *p* = .012, η_p_^2^ = .021. This pattern of results does not mirror those traditionally found in other-ethnicity studies, where there are opposite patterns in accuracy between white and non-white participants for white and non-white face items, suggesting that this test does not have an own-ethnicity effect. However, this may be due to the heterogeneity of both the non-white faces and the non-white participants used in this analysis. Because they are not from one ethnic group, we are unable to determine whether these differences are because other ethnicities are also included in this group, or whether the non-white faces were more difficult to recognise.

We repeated this analysis combining both online samples and found the same result. Participants were allocated into either the white participants (N = 17923) or non-white participants (N= 5689). The analysis was conducted using a 2x2 mixed factor ANOVA with Item Ethnicity (white, non-white) as the within-subjects factor and Participant Ethnicity (white, non-white) as the between-subjects factor. A significant interaction between factors suggested the presence of an own-ethnicity effect, F(1, 23610) = 153.39, p < .001, η_p_^2^ = .006. Simple main effects show that white participants performed 6.3% worse (CI_95_, -6.42 to -6.14) on non-white face items than white face items, F(1, 23610) = 7695.35, *p* < .001, η_p_^2^ = .246. However, non-white participants were also 4.5% worse (CI_95_, -4.72 to -4.23) on non-white face items than white face items, F(1, 23610) = 1239.56, *p* < .001, η_p_^2^ = .050.

While this pattern of results does not mirror those traditionally found in studies of the own-ethnicity effect as there is no cross-over pattern (e.g. [3,4]), the lower accuracy for both white and non-white participants non-white face items suggests that there is an own-ethnicity effect. However, to make confirm this a true cross-over study will be needed to effectively measure the full extent of the own-ethnicity effect in this test. To combat ethnicity effects across different test populations, we suggest that researchers make their own version of the UNSW Face Test and substitute the faces in the test with those from their target demographics. This would enable the identification of super-recognizers that demonstrate superiority for faces within the chosen demographic.

Analysis by participant gender

The effect of gender on accuracy on the UNSW Face Test was analysed using Online Samples 1 and 2. Gender comparisons show while there is no meaningful difference in overall accuracy between female and male participants (Females: 62.3%; Males: 61.8%), likely because the test includes equal numbers of female and male faces. The lack of difference between gender groups supports previous face identification research that also shows no difference between females and males [5]. However, there are some minor differences in the analysis of age effects by participant gender. Females do appear to peak in ability slightly earlier in age than males, at 30.3 (SE = 0.3) years and 31.4 years old (SE = 0.3) respectively.

References

1. McKone E, Wan L, Pidcock M, Crookes K, Reynolds K, Dawel A, et al. A critical period for faces: Other-race face recognition is improved by childhood but not adult social contact. Sci Rep. 2019;9(1):12820.

2. Meissner CA, Brigham JC. Thirty years of investigating the own-race bias in memory for faces: a meta-analytic review. Psychology, Public Policy, and Law. 2001;7(1):3.

3. Megreya AM, White D, Burton AM. The other-race effect does not rely on memory: Evidence from a matching task. The Quarterly Journal of Experimental Psychology. 2011;64(8):1473-83.

4. Wan L, Crookes K, Dawel A, Pidcock M, Hall A, McKone E. Face-blind for other-race faces: Individual differences in other-race recognition impairments. Journal of experimental psychology General. 2017;146(1):102-22.

5. Megreya AM, Bindemann M. Individual differences in personality and face identification. Journal of Cognitive Psychology. 2013;25(1):30-7.
