# Supplementary material for: UNSW Face Test: A screening tool for super-recognizers
Source: PLoS One. 2020 Nov 16;15(11):e0241747. doi: 10.1371/journal.pone.0241747 (PMC7668578; doi:10.1371/journal.pone.0241747)
Supplement: S2 Appendix — (DOCX) [file pone.0241747.s002.docx]

**UNSW Face Test: A screening tool for super-recognizers**

**S2 Appendix: Additional analysis**

James D. Dunn, Stephanie Summersby, Alice Towler, Josh P. Davis, and David White

*Corresponding Author: James D. Dunn (*[*j.d.dunn@unsw.edu.au*](mailto:j.d.dunn@unsw.edu.au)*)*

In the following analyses with examine the properties of the two sub-types of the UNSW Face Test: The Recognition memory task and the Match-to-sample sorting task. We repeat the main analyses performed in the manuscript for each sub-task. The data used for this analysis is also included in S1 Dataset.

Accuracy by sub-task

S2 Table 1 shows the mean accuracy, standard deviation and correlations between the memory and sort sub-task for each sample. Because the correlation was similar across all samples but because of differences in sample size, only the larger normative and two online samples were statistically significant (*p* < .05).

**S2 Table 1. Mean accuracy, standard deviations, and correlations for Memory and Sort task for each participant sample.**

|  | *n* | Memory Task | | Sort Task | | Pearson’s *r* |
| --- | --- | --- | --- | --- | --- | --- |
|  |  | *Mean (%)* | *SD (%)* | *Mean (%)* | *SD (%)* |  |
| Normative Sample | 290 | 60.1 | 9.0 | 58.3 | 6.7 | .187* |
| Online Sample 1 | 22,776 | 64.5 | 8.7 | 60.7 | 7.6 | .234** |
| Online Sample 2 | 836 | 66.6 | 8.9 | 63.6 | 7.9 | .233** |
| Lab Sample 1^✝^ | 80 | 60.2 | 7.9 | 60.2 | 6.9 | .186 |
| Lab Sample 2 | 102 | 62.0 | 9.0 | 59.5 | 7.1 | .101 |

^✝^ Time 1 accuracy * Significant at .05 level. ** Significant at .01 level.

Response latency by sub-task

S2 Table 2 shows the mean accuracy, standard deviation and correlations between the memory and sort sub-task for each sample. None of the correlations between time and accuracy were significant.

**S2 Table 2. Mean total response time during overall and for each sub-test, standard deviations, and correlations with accuracy by subtask for each participant sample.**

|  | Overall | | | Memory Task | | | Sort Task | | |
| --- | --- | --- | --- | --- | --- | --- | --- | --- | --- |
|  | *Mean (s)* | *SD (s)* | *r* | *Mean (s)* | *SD (s)* | *r* | *Mean (s)* | *SD (s)* | *r* |
| Normative Sample | 405.2 | 384.2 | -.135 | 155.7 | 352.4 | -.060 | 249.6 | 146.1 | -.082 |
| Online Sample 1 | 494.9 | 141.3 | -.007 | 157.4 | 183.5 | -.028 | 337.5 | 139.7 | -.002 |
| Online Sample 2 | 485.3 | 302.1 | -.012 | 152.4 | 101.8 | -.055 | 332.9 | 258.6 | -.008 |
| Lab Sample 1^✝^ | 313.3 | 82.2 | .055 | 116.3 | 42.8 | -.101 | 197.0 | 509 | .032 |
| Lab Sample 2 | 267.9 | 747.0 | .108 | 95.9 | 34.2 | .072 | 172.0 | 51 | .098 |

^✝^ Time 1 accuracy * Significant at .05 level. ** Significant at .01 level.

Signal detection theory analysis

S2 Table 3 shows the mean hit rate and false alarm rate for each sub-task and overall. We have also computed and report the non-parametric signal detection theory measures of sensitivity (A’) and criterion (B’’).

**S2 Table 3. Hit rate, false alarm rate (FAs), A’ and B’’ for each participant sample separated by sub-task.**

|  | **Overall** | | | | **Memory task** | | | | | **Sort task** | | | | |
| --- | --- | --- | --- | --- | --- | --- | --- | --- | --- | --- | --- | --- | --- | --- |
|  | Hits | FAs | A’ | B’’ | Hits | FAs | A’ | B’’ | Hits | | FAs | A’ | B’’ |  |
| Normative Sample | 0.572 | 0.395 | 0.648 | 0.012 | 0.569 | 0.368 | 0.654 | 0.048 | 0.574 | | 0.408 | 0.638 | -0.000 |  |
| Online Sample 1 | 0.623 | 0.383 | 0.690 | -0.004 | 0.634 | 0.343 | 0.716 | 0.028 | 0.618 | | 0.403 | 0.669 | -0.018 |  |
| Online Sample 2 | 0.644 | 0.352 | 0.723 | 0.008 | 0.638 | 0.306 | 0.742 | 0.054 | 0.647 | | 0.374 | 0.708 | -0.012 |  |
| Lab Sample 1^✝^ | 0.561 | 0.357 | 0.667 | 0.040 | 0.488 | 0.284 | 0.663 | 0.147 | 0598 | | 0.394 | 0.665 | -0.004 |  |
| Lab Sample 2 | 0.576 | 0.369 | 0.671 | 0.024 | 0.550 | 0.310 | 0.686 | 0.073 | 0.590 | | 0.399 | 0.656 | -0.005 |  |

^✝^ Time 1 accuracy

Test-retest reliability by sub-task

We also compared the test-retest reliability of the two sub-tasks of the UNSW Face Test using Lab Sample 1 (see Manuscript Method for full details). Test-retest reliability for Memory task was *r*(78) = 0.496, *p* < .001, CI_95_ [0.310, 0.645]), while test-retest reliability for Sort task was *r*(78) = 0.440, *p* < .001, CI_95_ [0.244, 0.601]).

Convergent validity

Next, we sought to establish convergent validity. We again compared the correlations of the two sub-tasks of the UNSW Face Test to the GFMT and CFMT+ using Lab Sample 1 (see Manuscript Method for full details). The Memory task was significantly correlated with the CFMT+, *r*(78) = 0.481, *p* < .001, CI_95_ [0.212, 0.634]), but not the GFMT, *r*(77) = 0.191, *p* = .091, CI_95_ [-0.031, 0.346]). Conversely, the Sort task was correlated equally with the CFMT+, *r*(78) = 0.399, *p* < .001, CI_95_ [0.197, 0.569]), and the GFMT, *r*(77) = 0.336, *p* = .002, CI_95_ [0.124, 0.519]). This pattern of correlation supports those shown by overall accuracy, in that the UNSW Face Test is more strongly associated with face memory than matching ability. This is unsurprising, given that both the memory and sort tasks rely on face memory ability, while only the sort task relies on face matching ability.

Divergent validity

Finally, we establish divergent validity for the two sub-tasks. Both the Memory and Sort task were significantly correlated with the CFMT+, Memory: *r*(102) = 0.231, *p* = .020, CI_95_ [0.038, 0.407]); Matching: *r*(102) = 0.232, *p* = .019, CI_95_ [0.039, 0.408]), but neither sub-task was associated with the CCMT, Memory: *r*(102) = 0.032, *p* = .752, CI_95_ [-0.164, 0.225]); Matching: *r*(102) = 0.021, *p* = .831, CI_95_ [-0.174, 0.215]), or MFFT, Memory: *r*(102) = 0.118, *p* = .237, CI_95_ [-0.078, 0.306]); Matching: *r*(102) = 0.101, *p* = .312, CI_95_ [-0.095, 0.290]).
