# Supplementary material for: UNSW Face Test: A screening tool for super-recognizers
Source: PLoS One. 2020 Nov 16;15(11):e0241747. doi: 10.1371/journal.pone.0241747 (PMC7668578; doi:10.1371/journal.pone.0241747)
Supplement: Attachment — Submitted filename: Review of _UNSW Face Test_.pdf [file pone.0241747.s003.pdf]

<u>Review of PONE-D-20-16507</u>

The authors report a novel test developed with the aim of providing a screening tool for identification of super-recognizers (SRs). Positive aspects of the manuscript concern (i) the authors' change of face regarding the need for more challenging tests (some they have used previously online are insufficiently sensitive); (ii) the use of ecologically valid material and the novel match-to-sample sorting task, which combines face memory, perceptual matching and pile sorting. While come concerns could theoretically be addressed (cf. additional concerns), due to several major methodological concerns we cannot recommend this manuscript for publication. We appreciate the overall goal, but do not believe that the reported findings warrant the claim that "mass online testing is the ideal solution to identify super-recognizers in the population". We hope the authors will find our comments constructive and remain at their disposal for questions.

Sincerely,

Meike Ramon and Jeff Nador

<u>Major critiques</u>

(1) <u>Lack of quality assurance</u>: The test represents an uncontrolled, online-available test, with insufficient measures implemented for quality assurance to mitigate artificial inflation or reduction of critical thresholds for super-recognizer identification. Two aspects that cannot be identified are **repeated participation and data generated by non-human respondents**. Additionally negligent is the situation that Ss are merely instructed to use a laptop or desktop, but that **mobile device usage is neither disabled**, **nor** (apparently) **considered in the analysis**, and **no form of screen calibration** is implemented. Further effects that cannot be ascertained relate to **partial data exclusion** (i.e., what happened to data from Ss who completed only the first task?) and the (unexplained) fixed task order (resulting in any correlations between measures taken beyond the first being potentially contaminated by order effects).

(2) <u>Test-theoretical limitations</u>: Screening tools require validation on a cohort of interest identified through independent *appropriate* measures - a prerequisite to ascertain false negative rate (which cannot be addressed via "confirmatory testing via standardized measures and more detailed cognitive testing"). As this is not accomplished here, the authors' reasoning is unjustified and circular (e.g. *"the UNSW Face Test is a valid and reliable test that is uniquely suited to screening for super-recognizers",* p.6, l.107ff.).

To be clear, the authors provide no details on how normative performance was established, only how normative data were *obtained*. Normative data should be treated as something of a ground truth against which to measure further samples, but it is unclear why this sample ought to be considered "normative". No ground truth about SRs was ascertained from it, and they were not retested to confirm their SR status, as this study's aims (creation of a screening tool) would suggest.

(3) <u>Stimulus material/selection</u>: The authors acknowledge the importance of considering the ethnic composition of a cohort. In stark contrast, as stimuli were taken from a database *"of 236 consenting undergraduate students"* (p.6, l.122f.), without information concerning the age composition, we must surmise that the stimulus set suffers from a severely restricted age range. This is problematic for the development of an ecologically valid SR screening tool given same-age performance biases.

<u>Additional concerns</u>

The manuscript appears conceptually fragmented, with sections providing at times inconsistent or apparently conflicting information, as detailed in the examples (1-3) below.

(1) <u>3-fold rationale for creation of another online test of face cognition</u>

<u>Referring to the CFMT+ and GFTM the authors state that</u>

a) *"super-recognizers typically achieve ceiling or near-ceiling accuracy on existing standardised tests"* (p.4, l.82f.)

b) *"existing standardised tests of face identification ability are unsuitable for online testing"* (p.4, l.62ff.)

c) both *"use highly standardised images and captured under optimal studio conditions ... do not reflect the challenge of real-world face identification"* (p.5, l. 101ff.).

<u>Regarding the above aspects, note that the authors</u>

a) state that their test *"enables researchers to apply stricter selection criteria that other available tests, which boosts the average accuracy of the individuals selected in subsequent testing."* (p.2, l.26ff.). They later *"propose that researchers and employers verify the super-recognizer status of those who score highly on the UNSW Face Test in controlled conditions using existing standardised tests, such as the CFMT and GFMT."* (p.4, l.73ff.)

   This logic is flawed; naturally, Ss identified with a more challenging tool would excel in less sensitive ones.

b) ***themselves* (continue to) use online versions of the CFMT and GFTM to identify super-recognizers**.

c) the authors **disregard existing tools that specifically uses ambient images** in the context of a well-established paradigm: the **Models Memory Test** (also available as an online version), **despite referring to this paper** in their manuscript (Bate et al., 2018; reference #25).

<u>(2) lack of conceptual precision</u>

- **The authors state that with the two comprising experiments, the UNSW** *"captures people with a general ability to identify faces, across **memory** and **matching tasks**"* (p.5, l. 88f). While the first experiment involves an old/new recognition paradigm, the latter at first glance appears to tap into perceptual skills. However, this is not actually the case. The second experiment is later described as a *"Match-to-sample sorting task... **combin[ing] immediate face memory, perceptual matching and pile sorting**"* (p.7, l.152f.).

  Thus, the UNSW test actually comprises two tests involving recognition memory as measured over different delay periods, and under different conditions.
- Further, throughout the manuscript the authors use the **term "face identification ability" to encompass any aspect of face cognition**. This is especially important in light of the legal implications of the term "identification" as used to refer to the tasks performed by forensic experts, as White and colleagues have described in their previous work, and have conceptually delineated e.g. in Ramon, Bobak & White (2019).

(3) Unwarranted claims / selective referencing of the literature

- *"individual differences [...] generalise from one face identification task to another [...] and represent a domain-specific cognitive skill that is dissociable from [...] visual object processing ability"* (p.3, l.36ff.)
  There is ample evidence suggesting the contrary to both aspects: work focusing on individual differences has provided evidence of performance across tasks of face cognition that can be unrelated (Bate et al., 2018; Bobak, Dowsett, et al., 2016), as well object processing abilities investigated in over 700 cases in developmental prosopagnosia (Geskin & Behrmann, 2018).
  Moreover, this stands in **direct conflict with other statements in the manuscript:**
  "Further, any single test provides an unreliable indication of face identification ability." (p.4, l.72); *"While these abilities may be dissociable to a limited extent (e.g. [2, 25, 30]), the high correlation between them suggests there is substantial overlap in these two abilities"* (p.5, l.94ff.)
- *"finding super-recognizers is difficult because they make up just 2-3% of the general population"* (p.3, l.49f.); *Face identification ability is normally distributed, and people at the very top end – 'super recognizers' – demonstrate extraordinary innate abilities"* (p.7, l.8f.).
  As White and colleagues have discussed (Ramon et al., 2019), the actual prevalence is yet to be determined; current estimate vary depending on the cutoffs and criteria applied; it is yet to be determined whether SRs form a special group in itself or are just the extremes of a continuum (cf. Young & Noyes, 2019).
- *"small sample sizes limit the statistical power of comparisons between super-recognizers and normative sample"* (p.3, l. 53ff.)
  We argue that large quantities of data collected under conditions where the impact of undesired nuisance variables is unknown (see major flaw (1)) are not the solution to all problems. In cognitive neuroscience and neuropsychology, there is a long tradition of carefully designed studies conducted in small, carefully curated cohorts of rare populations, which draw upon specifically developed statistical approaches.
- *"These abilities are employed to greater or lesser degrees in different professional tasks that super-recognizers have been recruited to perform. For example, in CCTV surveillance, super-recognizers monitor footage for faces they have committed to memory (e.g. [29]), whereas passport officers match photo-ID to unfamiliar travellers."* (P.5, l.90ff.)
  This creates the two misleading impressions, namely that SRs are (a) specifically recruited (authors reference #29) and widely used; (b) perform only memory tests, vs. passport officers who allegedly only make facial comparisons.
  (a) This is not the case; rather than psychometric testing employed for personnel selection, individuals *already within* a given organization have been referred to as SRs based on "anecdotal" peer-evaluation. Note also that the MET does not use SRs for recognition and identification (personal communication). To our knowledge and based on an assessment across international security agencies, only a small fraction is interested in this topic/actively pursues their deployment.
  (b) The potential areas of deployment are wide-ranging and specifically include perceptual image comparisons (Ramon & Rjosk, in press).

(4) Methods: opaque descriptions, and insufficient explanation/justification of decisions hindering replication

- Criteria used to select heterogeneous ethnicities for target faces, or how foils were rated as similar to particular targets are unclear.
- Both experiments contain different numbers of trials, and there is no rationale provided for taking the sum of correct answers across these two tasks. Is there a compelling reason to weight them differently? If so, it should be provided; if not, the metric should be unweighted.
- No compelling rationale for including only the two measures reported here. Neither experiment's performance on its own was correlated with non-facial tests of the same abilities, and the composite score was shown *not* to correlate with non-facial tests of these abilities. One would expect quite the opposite if "there is substantial overlap in these two abilities" (p.5, l.96).
- No analysis of response times is provided. Thus, any SRs identified by the UNSW face test may simply have been trading off speed for accuracy and vice versa. Particularly in an inventory where ceiling effects are not present (and the test is quite difficult), such trade-offs should be of great concern in validating a screening tool. Moreover, no description of instructions to participants was provided: were they ever explicitly instructed to focus on accuracy to the exclusion of speed?
- Granting that the authors *"intentionally did not calibrate the difficulty of the test so that mean accuracy was centred on the midpoint of the scale, as is common practice in standardised psychometric tests"* (p.4, l.78ff.), they still do not provide any information about how they *did* calibrate the test. Since they invite "researchers to create their own versions of the test" (p.6, l.117), this information is *absolutely crucial* to ensure replicability, as changing the stimulus set would obviously have the potential to alter any existing calibration. Likewise, calibration data for the two tasks contained in the UNSW face test were not provided for the normative cohort (or the subsequent samples).
